# Predictors of placebo response in three large clinical trials of the V1a receptor antagonist balovaptan in autism spectrum disorder

**DOI:** 10.1038/s41386-023-01573-9

**Published:** 2023-04-12

**Authors:** Russell Tobe, Yajing Zhu, Teresa Gleissl, Simona Rossomanno, Jeremy Veenstra-VanderWeele, Janice Smith, Eric Hollander

**Affiliations:** 1 Nathan S. Kline Institute for Psychiatric Research, Orangeburg, NY USA; 2 F. Hoffmann-La Roche Ltd., Welwyn Garden City, UK; 3 F. Hoffmann-La Roche Ltd., Basel, Switzerland; 4 Department of Psychiatry, Columbia University, New York, NY USA; 5 New York State Psychiatric Institute, New York, NY USA; 6 Department of Psychiatry and Behavioral Sciences and Albert Einstein College of Medicine, New York, NY USA

**Keywords:** Autism spectrum disorders, Drug development

## Abstract

High rates of placebo response are increasingly implicated in failed autism spectrum disorder (ASD) clinical trials. Despite this, there are limited investigations of placebo response in ASD. We sought to identify baseline predictors of placebo response and quantify their influence on clinical scales of interest for three harmonized randomized clinical trials of balovaptan, a V1a receptor antagonist. We employed a two-step approach to identify predictors of placebo response on the Vineland-II two-domain composite (2DC) (primary outcome and a caregiver measure) and Clinical Global Impression (CGI) scale (secondary outcome and a clinician measure). The initial candidate predictor set of variables pertained to participant-level, site-specific, and protocol-related factors. Step 1 aimed to identify influential predictors of placebo response using Least Absolute Shrinkage and Selection Operator (LASSO) regression, while Step 2 quantified the influence of predictors via linear regression. Results were validated through statistical bootstrapping approaches with 500 replications of the analysis dataset. The pooled participant-level dataset included individuals with ASD aged 5 to 62 years (mean age 21 [SD 10]), among which 263 and 172 participants received placebo at Weeks 12 and 24, respectively. Although no influential predictors were identified for CGI, findings for Vineland-II 2DC are robust and informative. Decreased placebo response was predicted by higher baseline Vineland-II 2DC (i.e., more advanced adaptive function), longer trial duration, and European (vs United States) sites, while increased placebo response was predicted by commercial (vs academic) sites, attention deficit hyperactivity disorder and depression. Identification of these factors may be useful in anticipating and mitigating placebo response in drug development efforts in ASD and across developmental and psychiatric conditions.

## Introduction

Autism spectrum disorder (ASD) is a common, lifelong, and heterogenous neurodevelopmental condition that is characterized by difficulties in social communication and interaction and repetitive/restrictive behaviors [1]. The experience of each autistic individual is unique in terms of clinical presentation, with varying symptom severity, associated symptoms, and comorbidities [1, 2]. Therefore, there is no one-size-fits-all approach for ASD therapies [2].

Despite ASD prevalence estimates as high as 1 in 44 [3], no studies have demonstrated conclusive pharmacotherapeutic efficacy in targeting core symptoms of either socialization and communication difficulties or restricted and repetitive behavior [4–8]. Accordingly, there are no US Food and Drug Administration (FDA)-approved pharmacotherapies for core symptoms [9], making ASD among the most prevalent health conditions lacking core medication treatments. This highlights an urgent need for ongoing efforts in development of novel pharmacotherapies, but there are several notable challenges associated with ASD clinical trial design and methodology [7]. First, the genetic architecture of ASD is heterogeneous, with contributions from both rare and common genetic variants [10], in addition to environmental factors [1]. Additionally, ongoing efforts to develop more sensitive, valid, and reliable outcome measures for assessing core ASD symptoms are needed [11, 12]. Current outcome measures are comprised either of subjective reporting by participants and their caregivers or clinician rater observations, which can introduce unintentional bias [13, 14].

Placebo response is a ubiquitous challenge in medicine. Though reported symptoms (e.g., pain, fatigue, and psychiatric symptoms) may be particularly vulnerable to expectancy bias and other forms of bias [15–17], placebo response has also been demonstrated in studies investigating more “objective” disease markers such as glycosylated hemoglobin in diabetes, hepatocyte histology in non-alcoholic steatohepatitis, body mass index (BMI), and blood pressure [18–21]. Unsurprisingly, high rates of placebo response have been observed across several ASD randomized controlled trials for multiple symptoms and endpoints [5, 7, 22–24]. By limiting the ability to discern treatment effects, placebo response may contribute to the low success rate of ASD trials and lack of approved pharmacotherapies [7]. Understanding sources of placebo response would therefore advance efforts to discern treatment effects of new pharmacotherapies desperately needed for at least a subgroup of autistic individuals [7, 24, 25]. While a few studies have used single samples or meta-analysis of published trials to assess potential predictors of placebo response, relatively small sample sizes and limited harmonization across studies has limited their impact [7, 24, 25]. These meta-analyses may be further limited by their use of study-level as opposed to participant-level data. Robust methodologies to identify predictors in larger samples are needed to better understand placebo response in ASD.

Balovaptan is a vasopressin 1a (V1a) receptor antagonist that has been investigated for the treatment of socialization and communication difficulties in autistic individuals. The balovaptan clinical development program comprised V1aduct, a phase 3 trial in adults, aV1ation, a phase 2 trial in children and adolescents, and VANILLA, a phase 2 trial in adults. The totality of evidence concluded that balovaptan did not show advantage over placebo in improving socialization and communication in ASD. Notably, a placebo response was observed across several primary and secondary endpoints, including the Vineland-II two-domain composite (2DC) (comprised of the Vineland-II Communication and Socialization domains) and the Clinical Global Impression (CGI) scale [5, 6, 8] (see supplement for details).

To harness the large sample size represented by these three harmonized trials, we employed a two-step statistical approach to robustly identify predictors of placebo response and to quantify their influence on clinical scales of interest in a participant-level dataset.

## Materials and methods

### Experimental procedures

This study followed the Strengthening the Reporting of Observational Studies in Epidemiology (STROBE) guideline for cohort studies. Ethics board approval was not required for this study as pooled anonymized data from clinical trials were used.

### Participants

Analyses comprised data from autistic individuals assigned to the placebo arm of the intention-to-treat population in three randomized controlled trials of balovaptan versus placebo in several global sites across North America and Europe. The trials included VANILLA (NCT01474278; 12 weeks; placebo arm *N* = 75 adults; 26 US sites; January 2014–May 2016) [6], V1aduct (NCT01793441; 24 weeks; placebo arm *N* = 158 adults; 46 North America and Europe sites; August 2018–July 2020) [5], and aV1ation (NCT02901431; 24 weeks; placebo arm *N* = 122 children and adolescents aged 5–17 years; 41 US sites; November 2016–September 2019) [8]. Other than age, full eligibility criteria for VANILLA, V1aduct, and aV1ation were generally similar [5, 6, 8].

**Fig. 1:**
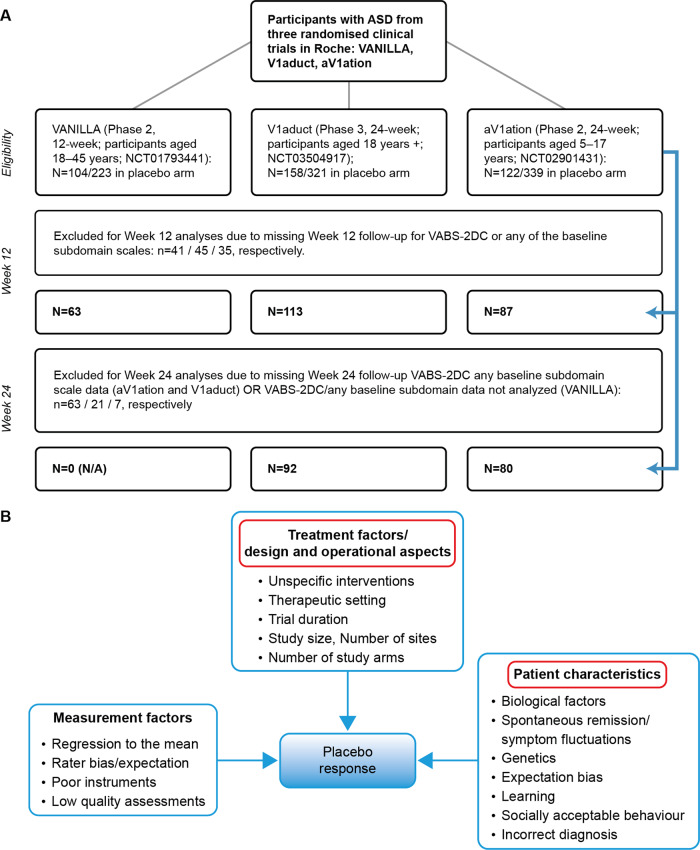
Methods for identification of predictors of placebo response. **A** Study population and (**B**) conceptualisation of the candidate predictors of placebo response. Adapted from and inspired by Rutherford and Roose, 2013 and Benedetti et al., 2011. Rutherford BR and Roose SP. A model of placebo response in antidepressant clinical trials. Am J Psychiatry. 2013;170:723–33. Benedetti F, Carlino E and Pollo A. How placebos change the patient’s brain. Neuropsychopharmacol. 2011;36:339–354. N/A not applicable.

To allow for the exploration of different predictor-endpoint relationships at different time points, Week 12 and 24 cohorts were created based on respective data. Both pooled cohort (aV1ation, V1aduct, and VANILLA participant data combined) and individual cohort data were analyzed to avoid missing cohort-specific signals. The main analyses comprised individuals with complete case analysis, whereby only participants with complete endpoint and baseline data of the included predictors were retained in the study. This led to a small reduction in sample size in both Week 12 and 24 cohorts (Fig. 1A). Missing data were not imputed because missing data are largely due to trial non-completion, which would not contribute to placebo response, and imputation of multiple correlated variables, if not thoroughly designed, may introduce bias into the analyses [26].

### Outcomes and covariates

The primary objective was to identify influential predictors of placebo response for Vineland-II 2DC change from baseline at Week 12 and Week 24 separately. The secondary objective was to identify predictors of placebo response for CGI – Improvement ≤3 (CGI-I; 3 = minimally improved, 2 = much improved, 1 = very much improved), at Week 12 and Week 24 separately.

The candidate predictor set included different dimensions from a conceptual model based upon previously identified factors that may influence placebo response (Fig. 1B) [7, 27]. In addition to basic demographic and site-specific factors, a broad range of candidate predictors were selected relating to symptom severity, diagnostic comorbidity, and family strain. Specifically, the candidate predictor set included baseline demographics (age, sex, intelligence quotient [IQ], BMI); the Social Responsiveness Score raw total score; CGI – Severity (see supplement for details): low severity (<5: mildly ill, moderately ill) versus high severity (markedly ill, severely ill, extremely ill); Vineland-II Socialization and Communication standard scores; Pediatric Quality of Life™ Inventory Family Impact total score; Repetitive Behavior Scale – Revised (RBS-R) baseline subscale scores (compulsive, restricted, ritualistic, sameness, self-injurious, stereotyped); concomitant medications; and comorbidities. In addition to number of sites per arm, individual site-specific factors included commercial versus academic, number of participants enrolled, and percentage dropout. Percentage dropout was a post-hoc predictor used as a proxy summary for unmeasurable or unobserved site-level characteristics.

### Statistical analysis

We used knowledge-based and data-driven approaches in this analysis. A two-step analysis was carried out. Step 1 (variable selection) selected influential predictors of placebo response among the candidate set of variables. In Step 2 (predictor significance), identified predictors were taken forward into linear regression analysis to quantify the size of the influence on placebo response. Analysis was repeated separately for Week 12 and 24 cohorts and for the pooled and individual cohorts across two endpoints (Vineland-II 2DC and CGI-I). All analyses used R version 3.1.4.

### Step 1: variable selection

To achieve robust findings, a number of methods were considered to identify influential predictors, including Least Absolute Shrinkage and Selection Operator (LASSO) [28], adding non-linear terms in LASSO regression (non-linear LASSO) [28], linear regression, and machine learning methods (random forest, neural networks) [29, 30]. Internal validation was performed with the bootstrap procedure with 500 replications [29–31]. All models were assessed on the following performance metrics: Root Mean Squared Error (RMSE), Mean Absolute Error (MAE), and R^2^ for the continuous outcome (Vineland-II 2DC). For the categorical outcome (CGI-I), specificity, sensitivity, and under the receiver-operating-curve (ROC) were considered. The best performing method was then selected, and the predictor was ranked based on magnitude of the coefficient. The most influential predictors, with absolute value of effect sizes greater than 1.96, were selected and passed to the next phase of analysis (Step 2) to quantify effect size. The entire analysis in Step 1 was performed for the pooled cohort and for each time point separately. In addition, to avoid missing potential predictors in individual datasets, the analysis was repeated for each study cohort separately for a total of 16 analyses (two endpoints [Vineland-II 2DC and CGI-I]; individual aV1ation, V1aduct, and VANILLA cohorts and a pooled cohort; and at two time points [Week 12 and Week 24]). All influential predictors across these analyses were collected as possible predictors to be included in Step 2.

### Step 2: predictor effect

Predictor effect was determined by quantifying the association between influential predictors and placebo response. Step 2 was necessary as estimates derived from penalized approaches used in Step 1 (e.g., LASSO) was by design, biased, as the algorithms prioritized predictive performance [28]. The relationship between the influential predictors and endpoints were instead evaluated using linear regression (change from baseline in Vineland-II 2DC and CGI-I) separately for each time point in the pooled cohort only. For completeness, results derived from the original model (including only the predictors specified in the trial protocol) and from the updated model (adding influential predictors) are reported. Only results of statistical significance are discussed (i.e., those corrected by Bonferroni testing for multiple comparisons).

## Results

### Baseline characteristics

The pooled participant-level data included autistic individuals aged 6 to 62 years (mean age 21 [SD 10]), among which 263 and 172 participants received placebo, and 405 and 248 received balovaptan at Week 12 and 24, respectively (Fig. 1A). Baseline characteristics were well balanced across balovaptan and placebo groups (Table 1A, B). Mean (SD) age in the Week 12 and 24 cohorts, respectively, was 21.7 (10.0) and 20.0 (10.1) in the placebo group and 20.8 (9.5) and 18.6 (9.9) in the balovaptan group. Baseline characteristics for the individual study cohorts (i.e., aV1ation, VANILLA, and V1aduct) are included in the Supplemental Materials (Tables S1–3). The distribution of comorbidities and concomitant medications for individual study cohorts are provided in the Supplemental Materials (Fig. S1).

**Table 1:** **A**. Baseline characteristics for balovaptan versus placebo groups in the Week 12 pooled cohort. Complete cases only. **B**. Baseline characteristics for balovaptan versus placebo groups in the Week 24 pooled cohort. Complete cases only.

	Balovaptan (*N* = 405)	Placebo (*N* = 263)	Total (*N* = 668)	*P* value
A				
Study, *n* (%)				
aV1ation				1.00
N	246 (60.7)	176 (66.9)	422 (63.2)	
Y	159 (39.3)	87 (33.1)	246 (36.8)	
VANILLA				1.00
N	285 (70.4)	200 (76.0)	485 (72.6)	
Y	120 (29.6)	63 (24.0)	183 (27.4)	
V1aduct				0.10
N	279 (68.9)	150 (57.0)	429 (64.2)	
Y	126 (31.1)	113 (43.0)	239 (35.8)	
Age				1.00
Mean (SD)	20.8 (9.5)	21.7 (10.0)	21.2 (9.7)	
Range	6.0–62.0	5.0–58.0	5.0–62.0	
IQ				1.00
Mean (SD)	99.5 (17.1)	101.9 (17.6)	100.5 (17.3)	
Range	70.0–143.0	70.0–175.0	70.0–175.0	
Sex, *n* (%)				
Female				1.00
N	356 (87.9)	226 (85.9)	582 (87.1)	
Y	49 (12.1)	37 (14.1)	86 (12.9)	
Male				1.00
N	49 (12.1)	37 (14.1)	86 (12.9)	
Y	356 (87.9)	226 (85.9)	582 (87.1)	
Country, *n* (%)				
Europe				1.00
N	258 (63.7)	179 (68.1)	437 (65.4)	
Y	147 (36.3)	84 (31.9)	231 (34.6)	
US and Canada				1.00
N	147 (36.3)	84 (31.9)	231 (34.6)	
Y	258 (63.7)	179 (68.1)	437 (65.4)	
Race, *n* (%)				
Asian				1.00
N	388 (95.8)	253 (96.2)	641 (96.0)	
Y	17 (4.2)	10 (3.8)	27 (4.0)	
Black/African American				1.00
N	384 (94.8)	248 (94.3)	632 (94.6)	
Y	21 (5.2)	15 (5.7)	36 (5.4)	
White				1.00
N	61 (15.1)	38 (14.4)	99 (14.8)	
Y	344 (84.9)	225 (85.6)	569 (85.2)	
BMI (baseline)				1.00
Mean (SD)	26.1 (7.2)	25.7 (7.7)	25.9 (7.4)	
Range	13.8–66.6	13.0–56.5	13.0–66.6	
Site weight by arm				<0.01
Mean (SD)	7.2 (3.6)	5.4 (3.7)	6.5 (3.7)	
Range	1.0–14.0	1.0–16.0	1.0–16.0	
Site number by arm				1.00
Mean (SD)	35.3 (7.3)	36.4 (8.7)	35.7 (7.9)	
Range	24.0–40.0	22.0–44.0	22.0–44.0	
PedsQL^**TM**^: Family Impact total (baseline)				1.00
Mean (SD)	59.2 (20.2)	58.5 (19.1)	58.9 (19.7)	
Range	0.0–100.0	0.0–100.0	0.0–100.0	
SRS-2: total raw (baseline)				1.00
Mean (SD)	117.7 (21.3)	120.3 (23.0)	118.7 (22.0)	
Range	66.0–186.0	67.0–184.0	66.0–186.0	
Vineland-II: 2DC (baseline)				1.00
Mean (SD)	68.0 (14.9)	67.4 (15.7)	67.8 (15.2)	
Range	20.5–102.5	20.5–104.0	20.5–104.0	
Vineland-II: Communication (baseline)				1.00
Mean (SD)	67.7 (17.6)	67.2 (19.0)	67.5 (18.2)	
Range	21.0–118.0	21.0–113.0	21.0–118.0	
Vineland-II: Socialization (baseline)				1.00
Mean (SD)	68.4 (16.0)	67.6 (15.8)	68.1 (15.9)	
Range	20.0–115.0	20.0–111.0	20.0–115.0	
RBS-R: compulsive (baseline)				1.00
Mean (SD)	4.3 (4.2)	4.2 (4.2)	4.3 (4.2)	
Range	0.0–22.0	0.0–21.0	0.0–22.0	
RBS-R: restricted (baseline)				1.00
Mean (SD)	3.4 (2.7)	3.3 (2.6)	3.3 (2.7)	
Range	0.0–12.0	0.0–12.0	0.0–12.0	
RBS-R: ritualistic (baseline)				1.00
Mean (SD)	4.6 (4.0)	4.7 (3.8)	4.6 (3.9)	
Range	0.0–18.0	0.0–17.0	0.0–18.0	
RBS-R: sameness (baseline)				1.00
Mean (SD)	7.8 (6.3)	8.4 (6.2)	8.0 (6.3)	
Range	0.0–31.0	0.0–29.0	0.0–31.0	
RBS-R: self-injurious (baseline)				1.00
Mean (SD)	2.2 (3.0)	2.1 (2.9)	2.2 (3.0)	
Range	0.0–16.0	0.0–19.0	0.0–19.0	
RBSR: stereotyped (baseline)				1.00
Mean (SD)	3.8 (3.5)	3.2 (3.0)	3.6 (3.3)	
Range	0.0–16.0	0.0–15.0	0.0–16.0	
Dropout by arm site				1.00
Mean (SD)	0.1 (0.1)	0.1 (0.1)	0.1 (0.1)	
Range	0.0–0.7	0.0–0.7	0.0–0.7	
Vineland-II: 2DC				1.00
Mean (SD)	3.8 (9.7)	3.8 (10.2)	3.8 (9.9)	
Range	−3.5 to 65.5	−30.5 to 40.0	−33.5 to 65.5	
Cognitive and attention disorders and disturbances, *n* (%)				1.00
N	220 (54.3)	143 (54.4)	363 (54.3)	
Y	185 (45.7)	120 (45.6)	305 (45.7)	
Anxiety disorders and symptoms, *n* (%)				1.00
N	260 (64.2)	163 (62.0)	423 (63.3)	
Y	145 (35.8)	100 (38.0)	245 (36.7)	
Depressed mood disorders and disturbances, *n* (%)				1.00
N	325 (80.2)	196 (74.5)	521 (78.0)	
Y	80 (19.8)	67 (25.5)	147 (22.0)	
Sleep disorders and disturbances, *n* (%)				1.00
N	319 (78.8)	215 (81.7)	534 (79.9)	
Y	86 (21.2)	48 (18.3)	134 (20.1)	
Developmental disorders NEC, *n* (%)				0.49
N	368 (90.9)	253 (96.2)	621 (93.0)	
Y	37 (9.1)	10 (3.8)	47 (7.0)	
Mood disorders and disturbances NEC, *n* (%)				1.00
N	376 (92.8)	246 (93.5)	622 (93.1)	
Y	29 (7.2)	17 (6.5)	46 (6.9)	
Psychiatric and behavioral symptoms, *n* (%)				1.00
N	400 (98.8)	261 (99.2)	661 (99.0)	
Y	5 (1.2)	2 (0.8)	7 (1.0)	
Manic and bipolar mood disorders and disturbances, *n* (%)				1.00
N	397 (98.0)	259 (98.5)	656 (98.2)	
Y	8 (2.0)	4 (1.5)	12 (1.8)	
Psychiatric disorders NEC, *n* (%)				1.00
N	395 (97.5)	259 (98.5)	654 (97.9)	
Y	10 (2.5)	4 (1.5)	14 (2.1)	
Impulse control disorders NEC, *n* (%)				1.00
N	400 (98.8)	259 (98.5)	659 (98.7)	
Y	5 (1.2)	4 (1.5)	9 (1.3)	
Disturbances in thinking and perception, *n* (%)				1.00
N	405 (100.0)	262 (99.6)	667 (99.9)	
Y	0 (0.0)	1 (0.4)	1 (0.1)	
Suicidal and self-injurious behaviors NEC, *n* (%)				1.00
N	400 (98.8)	261 (99.2)	661 (99.0)	
Y	5 (1.2)	2 (0.8)	7 (1.0)	
Schizophrenia and other psychotic disorders, *n* (%)				1.00
N	403 (99.5)	260 (98.9)	663 (99.3)	
Y	2 (0.5)	3 (1.1)	5 (0.7)	
Eating disorders and disturbances, *n* (%)				1.00
N	401 (99.0)	262 (99.6)	663 (99.3)	
Y	4 (1.0)	1 (0.4)	5 (0.7)	
Changes in physical activity, *n* (%)				1.00
N	396 (97.8)	261 (99.2)	657 (98.4)	
Y	9 (2.2)	2 (0.8)	11 (1.6)	
Personality disorders and disturbances in behavior, *n* (%)				1.00
N	396 (97.8)	261 (99.2)	657 (98.4)	
Y	9 (2.2)	2 (0.8)	11 (1.6)	
Adjustment disorders including subtypes, *n* (%)				1.00
N	403 (99.5)	262 (99.6)	665 (99.6)	
Y	2 (0.5)	1 (0.4)	3 (0.4)	
Communication disorders and disturbances, *n* (%)				<0.01
N	405 (100.0)	263 (100.0)	668 (100.0)	
Somatic symptom and related disorders, *n* (%)				1.00
N	405 (100.0)	262 (99.6)	667 (99.9)	
Y	0 (0.0)	1 (0.4)	1 (0.1)	
Antidepressants, *n* (%)				1.00
N	295 (72.8)	180 (68.4)	475 (71.1)	
Y	110 (27.2)	83 (31.6)	193 (28.9)	
Antipsychotic, *n* (%)				1.00
N	330 (81.5)	216 (82.1)	546 (81.7)	
Y	75 (18.5)	47 (17.9)	122 (18.3)	
Benzodiazepine, *n* (%)				1.00
N	392 (96.8)	251 (95.4)	643 (96.3)	
Y	13 (3.2)	12 (4.6)	25 (3.7)	
GABA B antagonist, *n* (%)				1.00
N	404 (99.8)	262 (99.6)	666 (99.7)	
Y	1 (0.2)	1 (0.4)	2 (0.3)	
Mood stabilizer anticonvulsants, *n* (%)				1.00
N	389 (96.0)	246 (93.5)	635 (95.1)	
Y	16 (4.0)	17 (6.5)	33 (4.9)	
Opiates, *n* (%)				<0.01
N	405 (100.0)	263 (100.0)	668 (100.0)	
Other anxiolytics, *n* (%)				1.00
N	289 (71.4)	192 (73.0)	481 (72.0)	
Y	116 (28.6)	71 (27.0)	187 (28.0)	
Sedatives, *n* (%)				1.00
N	405 (100.0)	262 (99.6)	667 (99.9)	
Y	0 (0.0)	1 (0.4)	1 (0.1)	
Stimulants, *n* (%)				1.00
N	293 (72.3)	184 (70.0)	477 (71.4)	
Y	112 (27.7)	79 (30.0)	191 (28.6)	
High CGI-S at baseline, *n* (%)				1.00
N	253 (62.5)	161 (61.2)	414 (62.0)	
Y	152 (37.5)	102 (38.8)	254 (38.0)	
Commercial site, *n* (%)				1.00
N	216 (53.3)	118 (44.9)	334 (50.0)	
Y	189 (46.7)	145 (55.1)	334 (50.0)	

	Balovaptan (*N* = 248)	Placebo (*N* = 172)	Total (*N* = 420)	*P* value
B				
Study, *n* (%)				
aV1ation				0.27
N	98 (39.5)	92 (53.5)	190 (45.2)	
Y	150 (60.5)	80 (46.5)	230 (54.8)	
VANILLA				0.01
N	248 (100.0)	172 (100.0)	420 (100.0)	
V1aduct				0.27
N	150 (60.5)	80 (46.5)	230 (54.8)	
Y	98 (39.5)	92 (53.5)	190 (45.2)	
Age				1.00
Mean (SD)	18.6 (9.9)	20.0 (10.1)	19.2 (10.0)	
Range	6.0–54.0	5.0–58.0	5.0–58.0	
IQ				1.00
Mean (SD)	99.8 (17.3)	102.9 (17.2)	101.1 (17.3)	
Range	70.0–140.0	70.0–144.0	70.0–144.0	
Sex, *n* (%)				
Female				1.00
N	206 (83.1)	141 (82.0)	347 (82.6)	
Y	42 (16.9)	31 (18.0)	73 (17.4)	
Male				1.00
N	42 (16.9)	31 (18.0)	73 (17.4)	
Y	206 (83.1)	141 (82.0)	347 (82.6)	
Country, *n* (%)				
Europe				1.00
N	234 (94.4)	158 (91.9)	392 (93.3)	
Y	14 (5.6)	14 (8.1)	28 (6.7)	
US and Canada				1.00
N	14 (5.6)	14 (8.1)	28 (6.7)	
Y	234 (94.4)	158 (91.9)	392 (93.3)	
Race, *n* (%)				
Asian				1.00
N	234 (94.4)	168 (97.7)	402 (95.7)	
Y	14 (5.6)	4 (2.3)	18 (4.3)	
Black/African American				1.00
N	238 (96.0)	165 (95.9)	403 (96.0)	
Y	10 (4.0)	7 (4.1)	17 (4.0)	
White				1.00
N	37 (14.9)	20 (11.6)	57 (13.6)	
Y	211 (85.1)	152 (88.4)	363 (86.4)	
BMI (baseline)				1.00
Mean (SD)	24.8 (7.7)	24.7 (8.1)	24.8 (7.9)	
Range	13.8–66.6	13.0–56.5	13.0–66.6	
Site weight by arm				0.03
Mean (SD)	6.9 (3.4)	6.0 (4.1)	6.5 (3.7)	
Range	1.0–14.0	1.0–16.0	1.0–16.0	
Site number by arm				1.00
Mean (SD)	40.0 (0.0)	40.7 (3.5)	40.3 (2.3)	
Range	40.0–40.0	37.0–44.0	37.0–44.0	
PedsQL^**TM**^: Family Impact total (baseline)				1.00
Mean (SD)	59.6 (18.6)	59.7 (17.8)	59.7 (18.3)	
Range	9.7–100.0	12.5–100.0	9.7–100.0	
SRS-2: total raw (baseline)				1.00
Mean (SD)	117.0 (21.6)	119.8 (23.4)	118.2 (22.4)	
Range	66.0–186.0	73.0–184.0	66.0–186.0	
Vineland-II: 2DC (baseline)				1.00
Mean (SD)	72.1 (12.1)	70.4 (14.4)	71.4 (13.1)	
Range	29.5–102.5	20.5–98.5	20.5–102.5	
Vineland-II: Communication (baseline)				1.00
Mean (SD)	72.2 (14.9)	71.0 (17.3)	71.7 (15.9)	
Range	21.0–118.0	21.0–113.0	21.0–118.0	
Vineland-II: Socialization (baseline)				1.00
Mean (SD)	72.1 (14.1)	69.7 (15.0)	71.1 (14.5)	
Range	20.0–115.0	20.0–100.0	20.0–115.0	
RBS-R: compulsive (baseline)				1.00
Mean (SD)	4.5 (4.2)	4.3 (4.1)	4.4 (4.2)	
Range	0.0–22.0	0.0–19.0	0.0–22.0	
RBS-R: restricted (baseline)				1.00
Mean (SD)	3.5 (2.8)	3.3 (2.7)	3.4 (2.7)	
Range	0.0–12.0	0.0–11.0	0.0–12.0	
RBS-R: ritualistic (baseline)				1.00
Mean (SD)	4.8 (3.9)	4.8 (4.0)	4.8 (3.9)	
Range	0.0–17.0	0.0–17.0	0.0–17.0	
RBS-R: sameness (baseline)				1.00
Mean (SD)	8.0 (6.3)	8.7 (6.4)	8.3 (6.4)	
Range	0.0–31.0	0.0–29.0	0.0–31.0	
RBS-R: self-injurious (baseline)				1.00
Mean (SD)	2.5 (3.3)	2.3 (3.1)	2.4 (3.2)	
Range	0.0–16.0	0.0–19.0	0.0–19.0	
RBS-R: stereotyped (baseline)				0.38
Mean (SD)	4.4 (3.5)	3.5 (3.1)	4.0 (3.3)	
Range	0.0–16.0	0.0–15.0	0.0–16.0	
Dropout by arm site				1.00
Mean (SD)	0.2 (0.2)	0.2 (0.2)	0.2 (0.2)	
Range	0.0–0.9	0.0–0.7	0.0–0.9	
Vineland-II: 2DC				0.87
Mean (SD)	3.4 (9.3)	6.1 (10.6)	4.5 (9.9)	
Range	−5 to 29	−19.5 to 48	−35 to 48	
Cognitive and attention disorders and disturbances, *n* (%)				1.00
N	105 (42.3)	80 (46.5)	185 (44.0)	
Y	143 (57.7)	92 (53.5)	235 (56.0)	
Anxiety disorders and symptoms, *n* (%)				1.00
N	144 (58.1)	103 (59.9)	247 (58.8)	
Y	104 (41.9)	69 (40.1)	173 (41.2)	
Depressed mood disorders and disturbances, *n* (%)				1.00
N	195 (78.6)	128 (74.4)	323 (76.9)	
Y	53 (21.4)	44 (25.6)	97 (23.1)	
Sleep disorders and disturbances, *n* (%)				1.00
N	179 (72.2)	137 (79.7)	316 (75.2)	
Y	69 (27.8)	35 (20.3)	104 (24.8)	
Developmental disorders NEC, *n* (%)				1.00
N	234 (94.4)	163 (94.8)	397 (94.5)	
Y	14 (5.6)	9 (5.2)	23 (5.5)	
Mood disorders and disturbances NEC, *n* (%)				1.00
N	230 (92.7)	161 (93.6)	391 (93.1)	
Y	18 (7.3)	11 (6.4)	29 (6.9)	
Psychiatric and behavioral symptoms NEC, *n* (%)				1.00
N	243 (98.0)	170 (98.8)	413 (98.3)	
Y	5 (2.0)	2 (1.2)	7 (1.7)	
Manic and bipolar disorders and disturbances, *n* (%)				1.00
N	241 (97.2)	170 (98.8)	411 (97.9)	
Y	7 (2.8)	2 (1.2)	9 (2.1)	
Psychiatric disorders NEC, *n* (%)				1.00
N	242 (97.6)	168 (97.7)	410 (97.6)	
Y	6 (2.4)	4 (2.3)	10 (2.4)	
Impulse control disorders NEC, *n* (%)				1.00
N	246 (99.2)	171 (99.4)	417 (99.3)	
Y	2 (0.8)	1 (0.6)	3 (0.7)	
Disturbances in thinking and perception, *n* (%)				0.01
N	248 (100.0)	172 (100.0)	420 (100.0)	
Suicidal and self-injurious behaviors NEC, *n* (%)				1.00
N	243 (98.0)	171 (99.4)	414 (98.6)	
Y	5 (2.0)	1 (0.6)	6 (1.4)	
Schizophrenia and other psychotic disorders, *n* (%)				1.00
N	248 (100.0)	171 (99.4)	419 (99.8)	
Y	0 (0.0)	1 (0.6)	1 (0.2)	
Eating disorders and disturbances, *n* (%)				1.00
N	245 (98.8)	171 (99.4)	416 (99.0)	
Y	3 (1.2)	1 (0.6)	4 (1.0)	
Changes in physical activity, *n* (%)				1.00
N	242 (97.6)	170 (98.8)	412 (98.1)	
Y	6 (2.4)	2 (1.2)	8 (1.9)	
Personality disorders and disturbances in behavior, *n* (%)				1.00
N	240 (96.8)	170 (98.8)	410 (97.6)	
Y	8 (3.2)	2 (1.2)	10 (2.4)	
Adjustment disorders including subtypes, *n* (%)				1.00
N	246 (99.2)	171 (99.4)	417 (99.3)	
Y	2 (0.8)	1 (0.6)	3 (0.7)	
Communication disorders and disturbances, *n* (%)				0.01
N	248 (100.0)	172 (100.0)	420 (100.0)	
Somatic symptom and related disorders, *n* (%)				0.01
N	248 (100.0)	172 (100.0)	420 (100.0)	
Antidepressants, *n* (%)				1.00
N	188 (75.8)	121 (70.3)	309 (73.6)	
Y	60 (24.2)	51 (29.7)	111 (26.4)	
Antipsychotic, *n* (%)				1.00
N	200 (80.6)	143 (83.1)	343 (81.7)	
Y	48 (19.4)	29 (16.9)	77 (18.3)	
Benzodiazepine, *n* (%)				1.00
N	240 (96.8)	165 (95.9)	405 (96.4)	
Y	8 (3.2)	7 (4.1)	15 (3.6)	
GABA B antagonist, *n* (%)				1.00
N	247 (99.6)	171 (99.4)	418 (99.5)	
Y	1 (0.4)	1 (0.6)	2 (0.5)	
Mood stabilizer anticonvulsants, *n* (%)				1.00
N	240 (96.8)	158 (91.9)	398 (94.8)	
Y	8 (3.2)	14 (8.1)	22 (5.2)	
Opiates, *n* (%)				0.01
N	248 (100.0)	172 (100.0)	420 (100.0)	
Other anxiolytics, *n* (%)				1.00
N	159 (64.1)	125 (72.7)	284 (67.6)	
Y	89 (35.9)	47 (27.3)	136 (32.4)	
Sedatives, *n* (%)				0.01
N	248 (100.0)	172 (100.0)	420 (100.0)	
Stimulants, *n* (%)				1.00
N	168 (67.7)	120 (69.8)	288 (68.6)	
Y	80 (32.3)	52 (30.2)	132 (31.4)	
High CGI-S at baseline, *n* (%)				1.00
N	153 (61.7)	112 (65.1)	265 (63.1)	
Y	95 (38.3)	60 (34.9)	155 (36.9)	
Commercial site, *n* (%)				1.00
N	107 (43.1)	62 (36.0)	169 (40.2)	
Y	141 (56.9)	110 (64.0)	251 (59.8)	

*P* values, which have been corrected for multiple calculations using the Bonferroni method, were calculated to indicate the difference between the balovaptan and placebo arms.

*2DC* two-domain composite, *BMI* body mass index, *CGI-S* Clinical Global Impression Scale – Severity, *GABA* gamma-aminobutyric, *IQ* intelligence quotient, *N* no, *NEC* not elsewhere classified, *PedsQL™* Pediatric Quality of Life™ Inventory, *RBS-R* Repetitive Behavior Scale – Revised, *SD* standard deviation, *SRS-2* Social Responsiveness Scale, 2nd Edition, *US* United States, *Y* yes.

### Distribution of outcomes and predictors

Fig. S2 shows the distribution of outcomes and predictors of participants in the placebo arm, separately for Week 12 and 24 analyses. We found that, apart from site-related variables, all clinical scales share similar distributions in Week 12 and 24, including the pattern of outliers (<5% of participants overall).

### Predictors of placebo response

#### Step 1: variable selection

For Vineland-II 2DC model comparison between linear regression, LASSO non-linear form, random forest, and LASSO for the pooled Week 12 and 24 cohorts is shown in Table 2. The LASSO method was shown to have the best performance compared with other models, due to best model performance in replications, smallest error (MAE, RMSE), relative highest fit (R^2^; although low for the study as a whole), and lower degree of variability of the fit statistics. The LASSO model was therefore used in Step 1 for robust variable selection. Results for CGI-I indicate poor fit (ROC = 0.5) and hence further model developments for CGI-I were not continued. Model comparison for CGI-I data is included in the Supplemental Materials (Fig. S3).

**Table 2:** Model comparison for the Vineland-II 2DC data in the Week 12 and 24 pooled cohorts.

Method	RMSE	R2	MAE	RMSE SD	Value R2 SD	MAE SD
Week 12						
LASSO	9.78	0.09	7.08	0.81	0.04	0.59
LASSO – Non-linear	9.87	0.09	7.15	0.84	0.05	0.61
Random forest	9.79	0.08	7.09	0.79	0.05	0.56
Linear regression	10.78	0.07	8.09	0.82	0.04	0.64
Week 24						
LASSO	10.04	0.12	7.58	0.96	0.06	0.68
LASSO – Non-linear	10.29	0.10	7.77	0.97	0.07	0.68
Random forest	10.08	0.11	7.67	0.92	0.06	0.68
Linear regression	12.05	0.07	9.33	1.16	0.06	0.93

*2DC* two-domain composite, *LASSO* Least Absolute Shrinkage and Selection Operator, *MAE* mean absolute error, *RMSE* root mean squared error, *SD* standard deviation.

**Fig. 2:**
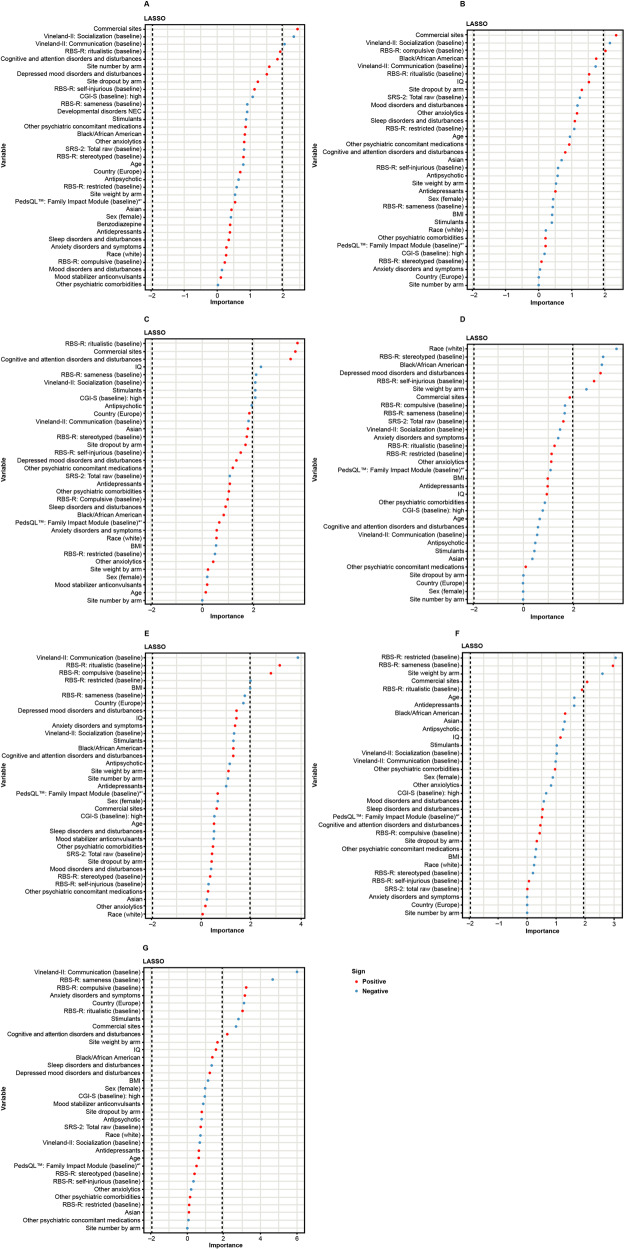
Predictors of change from baseline in Vineland-II 2DC. Influential predictors of placebo response were identified across cohorts (**A**) pooled Week 12 cohort, (**B**) aV1ation Week 12 cohort, (**C**) V1aduct Week 12 cohort, (**D**) VANILLA Week 12 cohort, (**E**) pooled Week 24 cohort, (**F**) aV1ation Week 24 cohort, (**G**) V1aduct Week 24 cohort. Red data points indicate increased placebo response. Blue data points indicate decreased placebo response. Dashed line indicates the cut-off at –1.96 and 1.96 for definition of an influential predictor. CGI-S Clinical Global Impression Scale – Severity, COM communication, EU Europe, PedsQL™ Pediatric Quality of Life™ Inventory, RBS-R Repetitive Behavior Scale – Revised, SOC socialization, VABS Vineland Adaptive Behavior Scales.

Figure 2 shows the identification of predictors of change from baseline in Vineland-II 2DC in the pooled Week 12 and 24 placebo cohorts and individual cohort analyses, ranked by effect sizes computed in LASSO. Across both the pooled and individual analyses, influential predictors of Vineland-II 2DC were identified in the following categories: (1) operations: site type (commercial versus academic), site size, site dropout rate (proxy for unmeasured site-related cohort and management heterogeneity); (2) baseline clinical scales: Vineland-II 2DC, RBS-R ritualistic, RBS-R compulsive, RBS-R self-injurious, RBS-R sameness, RBS-R restricted, CGI-I, IQ; (3) demographics: age, sex, country (United States [US]/Canada vs. Europe [EU]), BMI, race; (4) medical history (attention deficit hyperactivity disorder [ADHD], anxiety, and depression) and concomitant medication (stimulant use). No influential predictors for the CGI-I endpoint were identified (Fig. S4).

#### Step 2: predictor effect

The original predictors included in the respective trial protocols are baseline Vineland-II 2DC, age, sex, country, and IQ. The additional variables identified in Step 1 were added into the linear regression model in Step 2. Table 3 shows the estimates for the effects of predictors of the placebo response based upon the Vineland-II 2DC in the pooled placebo cohorts in Week 12 and 24.

**Table 3:** Predictor significance for change from baseline in Vineland-II 2DC in the placebo arm of the Week 12 and 24 cohorts.

Variable	Week 12			Week 24		
	*N*	Estimate (95% CI)	*P* value	*N*	Estimate (95% CI)	*P* value
Age	263	−0.05 (−0.19, 0.10)	0.527	172	0.01 (−0.19, 0.21)	0.92
Sex (Male)	37	0.83 (−2.57, 4.24)	0.630	141	1.37 (−2.60, 5.33)	0.50
Country (Europe)	179	−1.51 (−4.60, 1.58)	0.337	158	−6.57 (−12.73, −0.42)	**0.04**
Race						
Asian	10	2.58 (−5.31, 10.47)	0.520	4	−1.29 (−12.93, 10.35)	0.83
Black/African American	15	4.74 (−2.43, 11.91)	0.194	7	5.17 (−5.09, 15.44)	0.32
White	225	1.12 (−4.21, 6.44)	0.679	152	−0.56 (−7.42, 6.43)	0.87
IQ	263	−0.00 (−0.08, 0.07)	0.964	172	0.07 (−0.03, 0.17)	0.19
Baseline BMI	263	−0.03 (−0.20, 0.15)	0.766	172	−0.27 (−0.51, −0.03)	**0.02**
Site (commercial)	145	4.31 (1.48, 7.13)	**0.003**	110	1.11 (−2.85, 5.09)	0.58
Dropout	263	8.95 (0.55, 17.34)	**0.037**	172	1.82 (−7.26, 10.90)	0.69
Site weight by arm	263	−0.19 (−0.58, 0.20)	0.333	172	0.22 (−0.26, 0.70)	0.37
Cognitive and attention disorder and disturbances	120	3.71 (0.51, 6.92)	**0.023**	172	2.09 (−1.80, 5.98)	0.29
Anxiety disorders and symptoms	100	0.93 (−1.58, 3.44)	0.465	69	1.70 (−1.47, 4.87)	0.29
Depressed mood disorders and disturbances	67	4.50 (1.43, 7.57)	**0.004**	44	2.05 (−1.93, 6.04)	0.31
Stimulants	79	−2.08 (−5.38, 1.22)	0.215	52	−3.23 (−7.57, 1.11)	0.14
CGI-S (high; markedly ill, severely ill, extremely ill)	263	−2.39 (−4.84, 0.07)	0.057	172	−1.78 (−5.20, 1.64)	0. 31
Baseline Vineland-II 2DC	263	−0.23 (−0.31, −0.14)	**<0.001**	172	−0.32 (−0.44, −0.19)	**<0.001**
Baseline RBS-R: compulsive	263	0.10 (−0.35, 0.56)	0.650	172	0.78 (0.17, 1.40)	**0.01**
Baseline RBS-R: restricted	263	−0.38 (−1.06, 0.30)	0.270	172	−0.62 (−1.53, 0.29)	0.18
Baseline RBS-R: ritualistic	263	0.58 (0.05, 1.11)	**0.033**	172	0.71 (0.00, 1.42)	**0.05**
Baseline RBS-R: sameness	263	−0.21 (−0.57, 0.14)	0.241	172	−0.33 (−0.82, 0.16)	0.19
Baseline RBS-R: self-injurious	263	0.38 (−0.06, 0.83)	0.094	172	−0.20 (−0.76, 0.35)	0.46
Baseline RBS-R: stereotyped	263	0.24 (−0.25, 0.73)	0.334	172	0.09 (−0.51, 0.70)	0.76

Week 12 R^2^ = 18%; Week 24 R^2^ = 21%. Estimates, 95% CIs, and associated *P* values are derived using linear regression for the two cohorts separately, where the distribution of predictors are displayed side-by-side. The predictors that were included in the model were robustly selected from Step 1.

*2DC* two-domain composite, *BMI* body mass index, *CGI-S* Clinical Global Impressions – Severity, *CI* confidence interval, *IQ* intelligence quotient, *RBS-R* Repetitive Behavior Scale – Revised.

Bold values are statistically significant.

Increased placebo response at Week 12 was predicted by commercial (versus academic) sites (adjusted estimate 4.31; 95% CI: 1.48 to 7.13; *p* = 0.003), higher dropout rate per site (8.95; 0.55 to 17.34; *p* = 0.037), higher baseline RBS-R ritualistic (0.58; 0.05 to 1.11; *p* = 0.033), ADHD (3.71; 0.51 to 6.92; *p* = 0.023), and depression (4.50; 1.43 to 7.57; *p* = 0.004), and at Week 24, higher baseline RBS-R compulsive (0.78; 0.17 to 1.40; *p* = 0.01) and higher RBS-R ritualistic (0.71; 0.00 to 1.42; *p* = 0.05). Decreased placebo response at Week 12 was predicted by higher baseline Vineland-II 2DC (−0.22; −0.29 to −0.14; *p* = <0.001), and at Week 24, higher baseline Vineland-II 2DC (−0.32; −0.44 to −0.19; *p* = <0.001), from EU versus US (−6.57; −12.73 to −0.42; *p* = 0.04), and higher baseline BMI (−0.27; −0.51 to −0.03; *p* = 0.02). Adjusted R^2^ for Week 12 and 24 was 18% and 21%, respectively. Predictors were shown to have a less pronounced influence on placebo response at Week 24, compared with Week 12.

## Discussion

This study aimed to identify and quantify the influence of predictors of placebo response in three large harmonized clinical trials of balovaptan in ASD. High rates of placebo response can mask therapeutic signal detection and are increasingly implicated in failed ASD trials [5, 7, 8]. However, there are few investigations into placebo response in ASD trials and to our knowledge, only one has assessed participant-level data [7, 24, 25]. By leveraging participant-level data from three ASD multi-site trials of a single investigational medication across a large span of ages (ages 6–62 years), several participant, protocol, and site-related factors were found to influence placebo response on the primary outcome, Vineland-II 2DC.

For CGI-I, clinical response was considered as a CGI-I of 1 (very much improved) or 2 (much improved). For Vineland, cut-offs for minimally clinically important difference (MCID) for pediatric populations may vary by age. In adults, the Vineland-based MCID was set at 4 or 6 based on prior efforts and clinician consultation on MCID [32]. Approximately 18% of participants receiving placebo across the three studies reported a clinically significant response of a CGI-I score 1 or 2. In VANILLA 37.9% of adult participants receiving placebo met the MCID Vineland-II composite score criteria of ≥4 points at Week 12 and in V1aduct 48.5% met the MCID Vineland-II 2DC score of ≥6 points at Week 24 [5, 6]. Among participant-related factors, higher baseline Vineland-II 2DC, i.e., better adaptive functioning, was shown to reduce placebo response. This may be consistent with other studies that have demonstrated higher rates of placebo response with increased symptom severity in ADHD and hyperactivity associated with ASD [24, 33]. However, our finding of decreased placebo response in individuals with better adaptive functioning is notably in contrast to other investigations associating increased placebo response to lower symptom severity in studies of major depressive disorder, anxiety disorders, and several medical areas [34, 35]. This raises the possibility that individuals with higher baseline adaptive functioning may have less measurable room to improve upon already established adaptive skills [36]. Additionally, acquired skills at more advanced levels are complex and may require time courses for development beyond a 6-month trial duration. Because adaptive functions are a dimensional construct, raw scores on the Vineland-II 2DC vary by age and improve over time [37]. This may add noise to analyses of placebo response, at least with respect to adaptive functioning. A previous study assessing the use of citalopram for ASD identified that worse adaptive functioning (as measured by the Vineland-II Socialization domain only) predicted greater placebo response, a parallel to the results presented here [24].

Depression and ADHD comorbidities were shown in this study to be associated with increased placebo response. It is possible that individuals with comorbidity had positive past treatment experiences in management of their comorbidities that may have contributed to expectation bias and subsequently a higher placebo response [38]. Furthermore, participating in a clinical trial may encourage greater adherence to all concomitant medications, in addition to the active treatment, resulting in better outcomes. Though the presence of psychiatric comorbidity could simply be an indicator of higher overall impairment, it is notable that placebo response has been observed in both depression and ADHD randomized controlled trials. Importantly, depression is an episodic disorder more prone to spontaneous remissions [39, 40]. Depression and ADHD symptoms may also contribute to impairment in adaptive function, and improvements in these co-occurring conditions would be expected to manifest as better Vineland-II 2DC performance.

Higher BMI was shown to reduce placebo response in the Week 24, but not the Week 12, analysis cohort. BMI was previously shown not to be a significant predictor of placebo response in a meta-analysis of 86 randomized ASD pharmacologic or dietary supplement placebo-controlled trials [7]. However, as average BMI varies with age and sex, it is possible that differences in baseline characteristics between studies may have led to this inconsistency.

Higher baseline RBS-R ritualistic and RBS-R compulsive scores were associated with increased placebo response. These findings are unexpected, as we would anticipate that individuals with lower severity of ritualistic or compulsive behavior may be less resistant to change. In contrast, RBS-R sameness, RBS-R restricted, and RBS-R repetitive scores had no significant influence on placebo response. Another possible explanation may lie in the statistical properties of variables. Specifically, baseline RBS-R subscales are not correlated with other predictors, but correlations among the different RBS-R subscales were high (Fig. S5). This indicates that any statistical effects may be partially explained across the subscales. Further research will be required to understand the mechanistic and clinical reasons that underlie these findings. To our knowledge, no studies have identified a correlation between baseline severity on RBS-R scales and placebo response.

Among site/protocol-related factors, in line with previous literature [41, 42], we identified that placebo response is more likely at commercial versus academic sites. This could be due to the participant populations or the expertise at the respective sites [43]. Commercial sites may rely more heavily on study-specific recruitment efforts and advertisements, which may generate more expectation bias. Conversely, participants and their families at specialized academic sites may have more research familiarity, sophistication in understanding the importance of objective reporting, and hence be less prone to placebo response. Likewise, academic investigators may be more experienced in working with autistic individuals and may be better able to mitigate expectation bias [43]. We found that longer trial duration was predictive of a decreased placebo response, in agreement shorter trial duration has been predictive of high placebo responses in previous studies [16].

Consistent with findings in a meta-analysis of 421 anti-depressant trials [44], higher dropout rate per site across the balovaptan trials was associated with increased placebo response. Dropout rate was used as a post-hoc factor that acts as a proxy for unmeasurable features of site management and participant-related factors (e.g., expectation, heterogeneity, proximity to the site, etc.). It is possible that sites that better prevent dropout are also setting more modest expectations of potential benefit, leading to less disappointment if improvement is not seen. Sites with higher dropout rate may have set greater response expectations, leading both to higher placebo response and greater likelihood of participants dropping out if response is not seen. Participants who complete studies may also have stronger ties to the recruitment site and given their past experiences as patients and research participants, may be less prone to placebo effects. Similarly, in trials with a smaller dropout rate, the raters may be more familiar with the participant and better able to provide consistent and accurate ratings, particularly at study entry.

Fewer predictors and lower overall predictor effect were identified in the Week 24 versus Week 12 cohorts, suggesting that placebo response may be less likely in longer trials. This may indicate a learning curve for participants and their support providers in observing/reporting adaptive skills as measured by the Vineland-II 2DC. In parallel with our findings, shorter trials have been shown to increase placebo response in depression trials [45]; although this has not been previously observed as a predictive factor in ASD trials [7].

Interestingly, we observed that while both Vineland-II 2DC and CGI-I were subject to placebo response in the balovaptan trials, the predictors of placebo response identified in the Vineland-II 2DC were not replicated in the CGI-I. While the scales may share some similarities, the Vineland-II 2DC is specific to caregiver-reported adaptive functioning, compared with CGI, which assesses global improvement through clinician observations [32, 46]. Furthermore, due to the categorical structure of the CGI scale, modest improvements on the more dimensional Vineland-II 2DC may not be reflected in the CGI. Caregiver ratings have been previously reported to be more likely subject to placebo response than clinician ratings in ASD trials [7, 47]; however, another meta-analysis reported the opposite [25].

Participants’ treatment expectations are a known mediator of the placebo response [48]. Participants and their families in the aV1ation and V1aduct trials may have been impacted by press releases highlighting balovaptan’s FDA breakthrough therapy status [49]. Within the balovaptan trials, steps were taken to reduce treatment expectation such as specific participant, caregiver, and site training on placebo response (given by an independent provider), with the goal of managing expectations. Additionally, within aV1ation and V1aduct trials, central review and consistency checks of Vineland-II administration were performed, which may have mitigated placebo response. Prior studies have emphasized the importance of reducing placebo response by modifying the design of future trials [7, 47]. A recent meta-analysis of 122 major depressive disorder clinical trials indicated that adapting trial methodology to reduce placebo response, e.g., sequential parallel comparison design and placebo lead-in phases, may be less beneficial than focusing on other factors, such as enhanced participant selection, rater training, and treatment adherence monitoring [50]. The desired participant profile may vary depending on the outcomes and target symptoms being explored, requiring a balanced study-specific approach when considering participant-related factors. For example, a participant with higher symptom severity may have more room for improvement on the outcome measure of interest but greater probability of treatment resistance that could interfere with response.

An important potential modification to trial design includes the development of biomarkers as outcome measures [13, 51, 52]. Clinician- and caregiver-rated scales can be highly subjective, unintentionally biased, or fail to comprehensively assay symptoms, particularly in the case of heterogeneous conditions such as ASD [13, 53]. Therefore, it will be beneficial for objective, robust, and quantifiable outcome measures to be developed and utilized, including fine-grained observation of behavior such as eye tracking or biomarkers such as electroencephalography [12, 51]. Several initiatives are ongoing for the development of biomarkers for ASD, including the Autism Biomarkers Consortium for Clinical Trials [13]. Additionally, biomarkers may indicate differential neurobiological drivers that could be useful in enhancing participant selection based on known drug interactions [54].

Strengths of our analysis include that two of the studies had a long (6-month) duration, enabling rich analysis. The studies are also harmonized in their design (i.e., similar inclusion/exclusion criteria, common baseline measures, and outcome measures), enabling pooling of the participant-level data for a more robust analysis, as opposed to prior meta-analyses that compare study results [7, 25]. Furthermore, diversity in sites and inclusive entry criteria can be more readily generalized to a clinically identified autistic population. Robust methodology was also used for analysis, embracing both data-driven results and clinically driven insights, with the model and codes developed herein being fully reproducible and easily repeated or adapted by other researchers to identify placebo response predictors. In contrast to other efforts, we also reported operations and goodness-of-fit statistics [55].

Study limitations include the fact that evaluation of placebo response factors was not an a priori goal of the balovaptan trials. Greater expectation bias may be observed in trials of a later phase due to previous positive results. The pooled cohort comprises two phases of the balovaptan clinical development program (i.e., phase 2 aV1ation and VANILLA and phase 3 V1aduct), and greater expectation bias in the V1aduct trial may have skewed results in the direction of increased placebo response [5]. There were also an unequal set of sites in the US versus Europe, making it difficult to differentiate the impact of each region on placebo response. Furthermore, we were unable to assess all variables identified in the conceptual model (Fig. 1B) as some data were unavailable and/or challenging to quantify. In addition, the overall R^2^ for the analyses was low (18% for Week 12 analysis and 21% for the Week 24 analysis); however, this reflects the current limited understanding of placebo response in ASD. In line with this, there are a lack of ASD comparator studies, with none to date reporting R^2^ [7]. At present, there are no available datasets to test external validity of our results; however, this may be tested in the future as more research is carried out on placebo response. Finally, findings of the placebo response from Vineland-II 2DC may not be generalizable to other outcomes, including self-report measures.

## Conclusion

Our findings have identified several predictors of placebo response that can be further validated and ultimately considered for mitigating placebo response in future ASD trials. The application of our novel statistical methodology and associated findings may extend beyond ASD and contribute more broadly to psychiatric clinical trials. Better understanding of factors influencing placebo response may improve trial methods and lead to the development of efficacious therapies in ASD and other neuropsychiatric conditions.

## Supplementary information

Supplement

S1a

S1b

S2a

S2b

S3

## Data Availability

For up-to-date details on Roche’s Global Policy on the Sharing of Clinical Information and how to request access to related clinical study documents, see here: https://go.roche.com/data_sharing. Requests for data underlying this publication require a detailed, hypothesis-driven statistical analysis plan that is collaboratively developed by the requestor and company subject matter experts. Such requests should be directed to datarequest.autism@roche.com for consideration. Anonymized records for individual patients across more than one data source external to Roche cannot, and should not, be linked due to a potential increase in risk of patient re-identification.

## References

[1] Hodges H, Fealko C, Soares N (2020). Autism spectrum disorder: definition, epidemiology, causes, and clinical evaluation. Transl Pediatr.

[2] Malik-Soni N, Shaker A, Luck H, Mullin AE, Wiley RE, Lewis MES (2021). Tackling healthcare access barriers for individuals with autism from diagnosis to adulthood. Pediatr Res.

[3] Maenner MJ, Shaw KA, Baio J, Washington A, Patrick M, DiRienzo M (2020). Prevalence of autism spectrum disorder among children aged 8 years - autism and developmental disabilities monitoring network, 11 sites, United States, 2016. MMWR Surveill Summ.

[4] Thom RP, Pereira JA, Sipsock D, McDougle CJ (2021). Recent updates in psychopharmacology for the core and associated symptoms of autism spectrum disorder. Curr Psychiatry Rep.

[5] Jacob S, Veenstra-VanderWeele J, Murphy D, McCracken J, Smith J, Sanders K (2022). Efficacy and safety of balovaptan for socialisation and communication difficulties in autistic adults in North America and Europe: a phase 3, randomised, placebo-controlled trial. Lancet Psychiatry.

[6] Bolognani F, Del Valle Rubido M, Squassante L, Wandel C, Derks M, Murtagh L (2019). A phase 2 clinical trial of a vasopressin V1a receptor antagonist shows improved adaptive behaviors in men with autism spectrum disorder. Sci Transl Med.

[7] Siafis S, Çiray O, Schneider-Thoma J, Bighelli I, Krause M, Rodolico A (2020). Placebo response in pharmacological and dietary supplement trials of autism spectrum disorder (ASD): systematic review and meta-regression analysis. Mol Autism.

[8] Hollander E, Jacob S, Jou R, McNamara N, Sikich L, Tobe R (2022). Balovaptan vs placebo for social communication in childhood autism spectrum disorder: a randomized clinical trial. JAMA Psychiatry.

[9] Siafis S, Çiray O, Wu H, Schneider-Thoma J, Bighelli I, Krause M (2022). Pharmacological and dietary supplement treatments for autism spectrum disorder: a systematic review and network meta-analysis. Mol Autism.

[10] Masi A, DeMayo MM, Glozier N, Guastella AJ (2017). An overview of autism spectrum disorder, heterogeneity and treatment options. Neurosci Bull.

[11] Brugha TS, Doos L, Tempier A, Einfeld S, Howlin P (2015). Outcome measures in intervention trials for adults with autism spectrum disorders; a systematic review of assessments of core autism features and associated emotional and behavioural problems. Int J Methods Psychiatr Res.

[12] Anagnostou E, Jones N, Huerta M, Halladay AK, Wang P, Scahill L (2015). Measuring social communication behaviors as a treatment endpoint in individuals with autism spectrum disorder. Autism.

[13] McPartland JC, Bernier RA, Jeste SS, Dawson G, Nelson CA, Chawarska K (2020). The Autism Biomarkers Consortium for Clinical Trials (ABC-CT): scientific context, study design, and progress toward biomarker qualification. Front Integr Neurosci.

[14] Moricke E, Buitelaar JK, Rommelse NNJ (2016). Do we need multiple informants when assessing autistic traits? The degree of report bias on offspring, self, and spouse ratings. J Autism Dev Disord.

[15] Roji R, Stone P, Ricciardi F, Candy B (2020). Placebo response in trials of drug treatments for cancer-related fatigue: a systematic review, meta-analysis and meta-regression. BMJ Support Palliat Care.

[16] Weimer K, Colloca L, Enck P (2015). Placebo effects in psychiatry: mediators and moderators. Lancet Psychiatry.

[17] Duarte GS, Mainoli B, Rodrigues FB, Rato F, Machado T, Ferreira JJ, et al. Placebo response in chronic peripheral neuropathic pain trials: systematic review and meta-analysis. medRxiv. 2022.02.18.22271196.

[18] Lin C, Cai X, Yang W, Lv F, Nie L, Ji L (2020). Age, sex, disease severity, and disease duration difference in placebo response: implications from a meta-analysis of diabetes mellitus. BMC Med.

[19] Wilhelm M, Winkler A, Rief W, Doering BK (2016). Effect of placebo groups on blood pressure in hypertension: a meta-analysis of beta-blocker trials. J Am Soc Hypertens.

[20] Chin YH, Ng CH, Chew NW, Kong G, Lim WH, Tan DJH (2022). The placebo response rate and nocebo events in obesity pharmacological trials. A systematic review and meta-analysis. EClinicalMedicine.

[21] Han MAT, Altayar O, Hamdeh S, Takyar V, Rotman Y, Etzion O (2019). Rates of and factors associated with placebo response in trials of pharmacotherapies for nonalcoholic steatohepatitis: systematic review and meta-analysis. Clin Gastroenterol Hepatol.

[22] Aman MG, Findling RL, Hardan AY, Hendren RL, Melmed RD, Kehinde-Nelson O (2017). Safety and efficacy of memantine in children with autism: randomized, placebo-controlled study and open-label extension. J Child Adolesc Psychopharmacol.

[23] Yamasue H, Okada T, Munesue T, Kuroda M, Fujioka T, Uno Y (2020). Effect of intranasal oxytocin on the core social symptoms of autism spectrum disorder: a randomized clinical trial. Mol Psychiatry.

[24] King BH, Dukes K, Donnelly CL, Sikich L, McCracken JT, Scahill L (2013). Baseline factors predicting placebo response to treatment in children and adolescents with autism spectrum disorders: a multisite randomized clinical trial. JAMA Pediatr.

[25] Masi A, Lampit A, Glozier N, Hickie IB, Guastella AJ (2015). Predictors of placebo response in pharmacological and dietary supplement treatment trials in pediatric autism spectrum disorder: a meta-analysis. Transl Psychiatry.

[26] Hughes RA, Heron J, Sterne JAC, Tilling K (2019). Accounting for missing data in statistical analyses: multiple imputation is not always the answer. Int J Epidemiol.

[27] Rutherford BR, Roose SP (2013). A model of placebo response in antidepressant clinical trials. Am J Psychiatry.

[28] Tibshirani R (1996). Regression shrinkage and selection via the lasso. J R Stat Soc Ser B..

[29] Harrell Jr FE. Regression modeling strategies with applications to linear models, logistic and ordinal regression, and survival analysis. 2nd ed. Springer Cham: New York, NY; 2015.

[30] James G, Witten D, Hastie T, Tibshirani R. An introduction to statistical learning with applications in R. Springer: New York, NY; 2013.

[31] Collins GS, Reitsma JB, Altman DG, Moons KGM (2015). Transparent reporting of a multivariable prediction model for individual prognosis or diagnosis (TRIPOD): the TRIPOD statement. BMJ..

[32] Chatham CH, Taylor KI, Charman T, Liogier D’ardhuy X, Eule E, Fedele A (2018). Adaptive behavior in autism: minimal clinically important differences on the Vineland-II. Autism Res.

[33] Faraone SV, Newcorn JH, Cipriani A, Brandeis D, Kaiser A, Hohmann S (2022). Placebo and nocebo responses in randomised, controlled trials of medications for ADHD: a systematic review and meta-analysis. Mol Psychiatry.

[34] Stein DJ, Baldwin DS, Dolberg OT, Despiegel N, Bandelow B (2006). Which factors predict placebo response in anxiety disorders and major depression? An analysis of placebo-controlled studies of escitalopram. J Clin Psychiatry.

[35] Weimer K, Colloca L, Enck P (2015). Age and sex as moderators of the placebo response – an evaluation of systematic reviews and meta-analyses across medicine. Gerontology..

[36] Weimer K, Gulewitsch MD, Schlarb AA, Schwille-Kiuntke J, Klosterhalfen S, Enck P (2013). Placebo effects in children: a review. Pediatr Res.

[37] Pathak M, Bennett A, Shui AM (2019). Correlates of adaptive behavior profiles in a large cohort of children with autism: The Autism Speaks Autism Treatment Network registry data. Autism.

[38] Williams JB, Popp D, Kobak KA, Detke MJ (2012). P-640 - The power of expectation bias. Eur Psychiatry.

[39] Castells X, Saez M, Barcheni M, Cunill R, Serrano D, López B (2022). Placebo response and its predictors in attention deficit hyperactivity disorder: a meta-analysis and comparison of meta-regression and MetaForest. Int J Neuropsychopharmacol.

[40] Jones BD, Weissman CR, Razza LB, Ishrat Husain M, Brunoni AR, Daskalakis ZJ (2021). Protocol for a systematic review and meta-analysis of the placebo response in treatment-resistant depression: comparison of multiple treatment modalities. BMJ Open.

[41] Fraguas D, Diaz-Caneja CM, Pina-Camacho L, Umbricht D, Arango C (2019). Predictors of placebo response in pharmacological clinical trials of negative symptoms in schizophrenia: a meta-regression analysis. Schizophr Bull.

[42] Dobson ET, Strawn JR (2016). Placebo response in pediatric anxiety disorders: implications for clinical trial design and interpretation. J Child Adolesc Psychopharmacol.

[43] Jacob S, Anagnostou E, Hollander E, Jou R, McNamara N, Sikich L (2022). Large multicenter randomized trials in autism: key insights gained from the balovaptan clinical development program. Mol Autism.

[44] Salanti G, Chaimani A, Furukawa TA, Higgins JPT, Ogawa Y, Cipriani A (2018). Impact of placebo arms on outcomes in antidepressant trials: systematic review and meta-regression analysis. Int J Epidemiol.

[45] Furukawa TA, Cipriani A, Atkinson LZ, Leucht S, Ogawa Y, Takeshima N (2016). Placebo response rates in antidepressant trials: a systematic review of published and unpublished double-blind randomised controlled studies. Lancet Psychiatry.

[46] Busner JT, Targum SD (2007). The Clinical Global Impressions scale. Psychiatry (Edgmont).

[47] Jones RM, Carberry C, Hamo A, Lord C (2017). Placebo‐like response in absence of treatment in children with autism. Autism Res..

[48] Frisaldi E, Shaibani A, Benedetti F (2017). Why we should assess patients’ expectations in clinical trials. Pain Ther.

[49] FDA News. Roche’s autism drug balovaptan granted breakthrough therapy designation. 2018. https://www.fdanews.com/articles/185437-roches-autism-drug-balovaptan-granted-breakthrough-therapy-designation. Accessed February 24 2023.

[50] Whitlock ME, Woodward PW, Alexander RC (2019). Is high placebo response really a problem in depression trials? A critical re-analysis of depression studies. Innov Clin Neurosci.

[51] Klin A (2018). Biomarkers in autism spectrum disorder: challenges, advances, and the need for biomarkers of relevance to public health. Focus (Am Psychiatr Publ).

[52] Frye RE, Vassall S, Kaur G, Lewis C, Karim M, Rossignol D (2019). Emerging biomarkers in autism spectrum disorder: a systematic review. Ann Transl Med.

[53] Bangerter A, Manyakov NV, Lewin D, Boice M, Skalkin A, Jagannatha S (2019). Caregiver daily reporting of symptoms in autism spectrum disorder: observational study using web and mobile apps. JMIR Ment Health.

[54] Jensen AR, Lane AL, Werner BA, McLees SE, Fletcher TS, Frye RE (2022). Modern biomarkers for autism spectrum disorder: future directions. Mol Diagn Ther.

[55] Gromova M, Vaggelas A, Dallmann G, Seimetz D. Biomarkers: opportunities and challenges for drug development in the current regulatory landscape. Biomark Insights. 2020;15:1177271920974652. 10.1177/1177271920974652 PMC7727038 33343195

