# Supplementary material for: Predictors of placebo response in three large clinical trials of the V1a receptor antagonist balovaptan in autism spectrum disorder
Source: Neuropsychopharmacology. 2023 Apr 12;48(8):1201–16. doi: 10.1038/s41386-023-01573-9 (PMC10267133; doi:10.1038/s41386-023-01573-9)
Supplement: Supplementary file 1 — Supplement [file 41386_2023_1573_MOESM1_ESM.docx]

**Supplement to: Predictors of placebo response in three large clinical trials of the V1a receptor antagonist balovaptan in autism spectrum disorder**

Russell Tobe, Yajing Zhu, Teresa Gleissl, Simona Rossomanno, Jeremy Veenstra-Vanderweele, Janice Smith, Eric Hollander

**Clinical Global Impression Scale – Severity (CGI-S) category grouping**

Grouping of the CGI-S categories were based primarily on the distributional properties of the data, to avoid skewing the results by having categories that were too small. In each trial population, “moderately ill” included the largest share of the population (around 60%) while “markedly ill" (30%) included the second largest share. Grouping these two populations together or keeping each separate would have resulted in an overly small size for the remaining categories. Therefore, we split these two large categories and combined them with the adjacent closely related categories whilst still considering clinical interpretations.

**Summary of changes in Vineland-II two-domain composite (2DC) and CGI – Improvement (CGI-I) in the balovaptan trials**

In VANILLA the Vineland-II 2DC was not used, treatment differences between balovaptan and placebo increased with dose and were driven by improvements on the Vineland-II scores for the Socialization and Communication domains [1]. A post-hoc analysis of VANILLA participants

using a composite measure of the Vineland-II 2DC showed significant improvements with balovaptan 10 mg versus placebo [2]. In V1aduct and aV1ation at Weeks 12 and 24 no significant improvement in the balovaptan group compared with placebo were seen in change from baseline in Vineland-II 2DC score [3, 4]. In V1aduct there was a larger than expected placebo effect in change from baseline in Vineland-II 2DC score at Week 24 with 41.4% of the balovaptan group participants reporting an improvement of ≥6 points compared with 48.5% of the placebo group [3]. In aV1ation there were no differences in change from baseline on the Vineland-II 2DC, balovaptan (adjusted least-squares mean [LSM], 2.17; standard error [SE], 1.11) compared with placebo (LSM, 2.34; SE, 1.15) at Week 24 [4]. In V1aduct no consistent treatment effect was observed on the CGI-I scales, although a numerically higher percentage of responders defined by CGI-I criteria (scores of 1 or 2) was observed in all three balovaptan doses compared with placebo at Week 12 [1]. In V1aduct a considerable proportion of participants in the placebo group reported improvements in CGI-I at Week 12 and Week 24 [3]. In aV1ation at Weeks 12 and 24, there were no differences in the proportion of responders defined by CGI-I criteria between balovaptan and placebo [4].

**Tables**

**Table S1.** Baseline characteristics of participants in the balovaptan and placebo arms of the V1aduct (a) Week 12 and (b) Week 24 cohorts.

**Table S2.** Baseline characteristics of participants in the balovaptan and placebo arms of the aV1ation (a) Week 12 and (b) Week 24 cohorts.

**Table S3.** Baseline characteristics of participants in the balovaptan and placebo arms of the VANILLA Week 12 cohort.

**Figures**


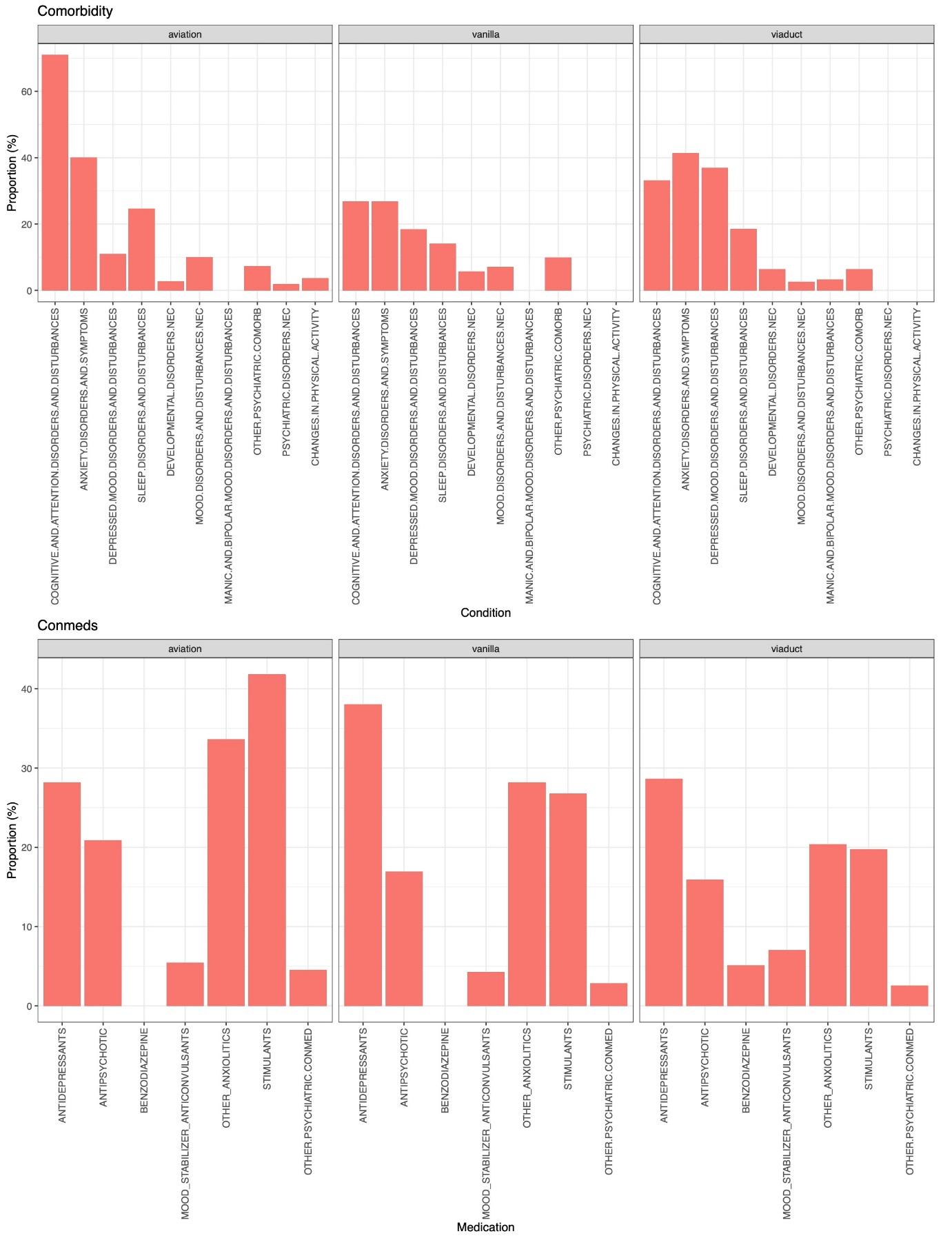


**Figure S1.** Distributions of comorbidities and concomitant medications in the placebo arms of the aV1ation, VANILLA, and V1aduct study cohorts. Comorbidities experienced by less than 10 individuals were grouped in the “Others” category. Concomitant medications taken by less than 10 individuals were grouped in the “Others” category. NEC not elsewhere classified.


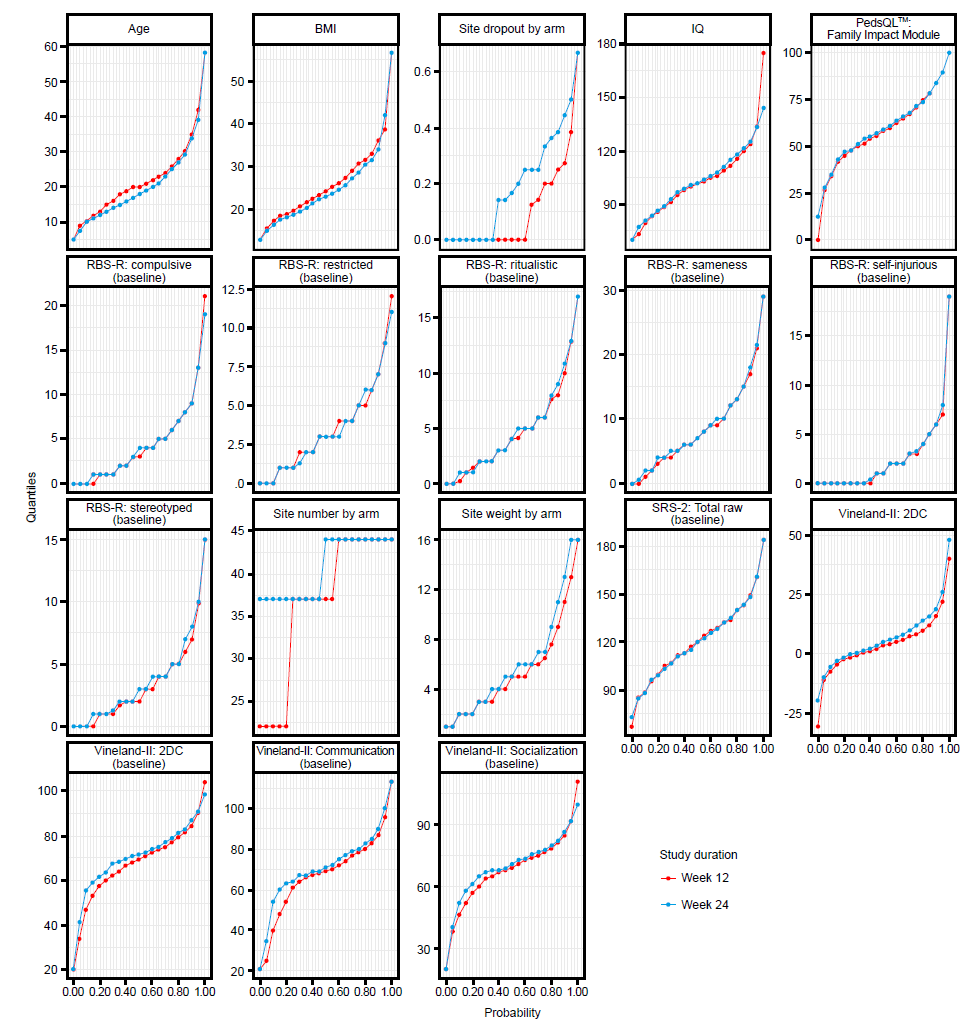


**Figure S2.** Distributions of outcomes and predictors by study in the Week 12 and Week 24 pooled placebo cohorts showing the behavior of corresponding cohorts, longitudinally. Only complete cases were included therefore Week 12 and Week 24 cohorts are disparate. This figure shows the alignment (same shape and trajectory) of the red (Week 12) and blue (Week 24) curves in the majority of the scales, indicating similar behavior of patients. Contrast scales with less alignment of curves highlight specific behavior differences of patients, e.g., site dropout by arm shows a different dropout pattern. VABS_2DC indicates the plot for “CFB in VABS_2DC”. “VABS_2DC_baseline” indicates baseline distribution. Complete cases only. 2DC two-domain composite, BBMI baseline BMI, BMI body mass index, CFB change from baseline, COM communication, SOC socialization, PedsQL Pediatric Quality of Life Inventory, RBS-R Repetitive Behavior Scale – Revised, SRS-2 Social Responsiveness Scale, 2^nd^ edition, VABS Vineland Adaptive Behavior Scales.


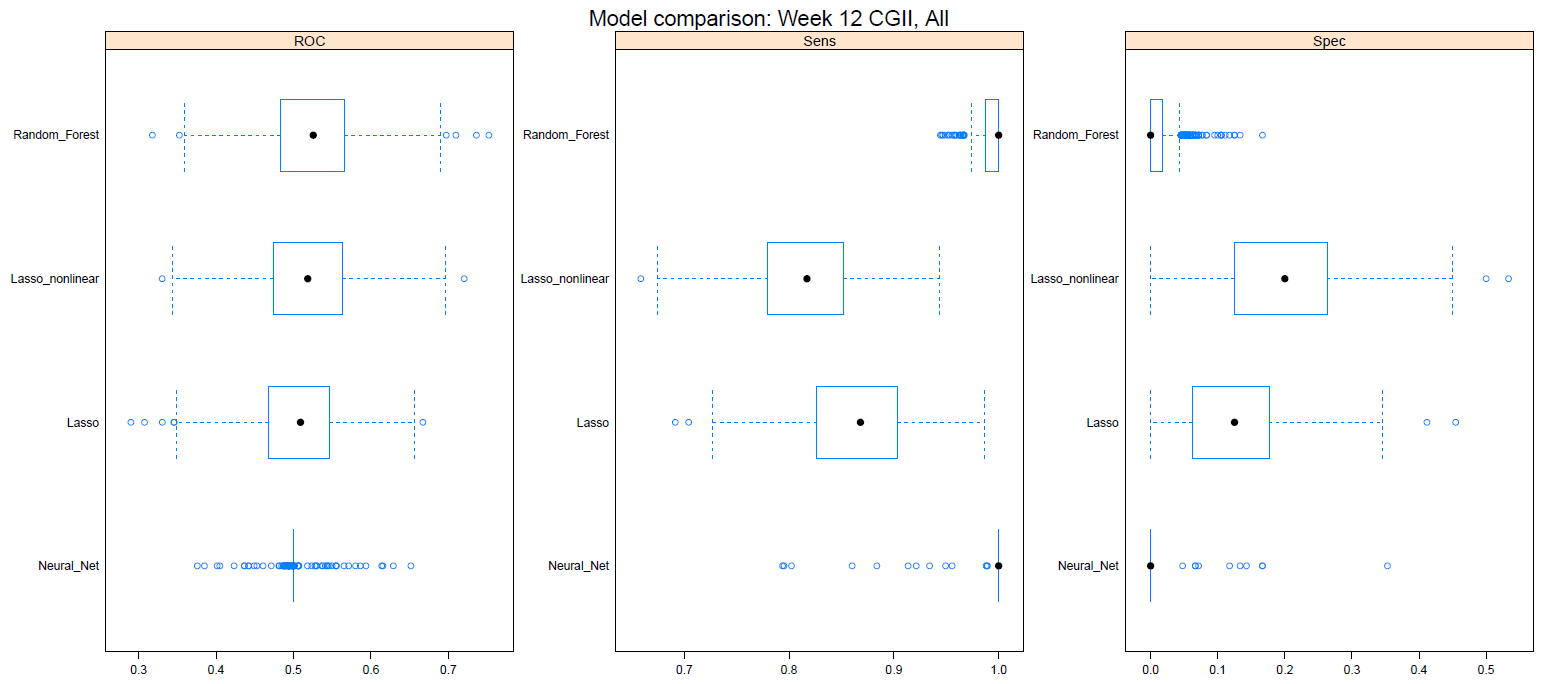


**Figure S3.** Performance of candidate models on Step 1 (predictor selection) for CGI-I at Week 12. Highly similar results were observed at Week 24; Week 24 results are not reported, to avoid repetitive data presentation. ROC value was close to 0.5, indicating poor fit. CGI-I Clinical Global Impressions Scale – Improvement, ROC area under the receiver operating curve, spec specificity, sens sensitivity.


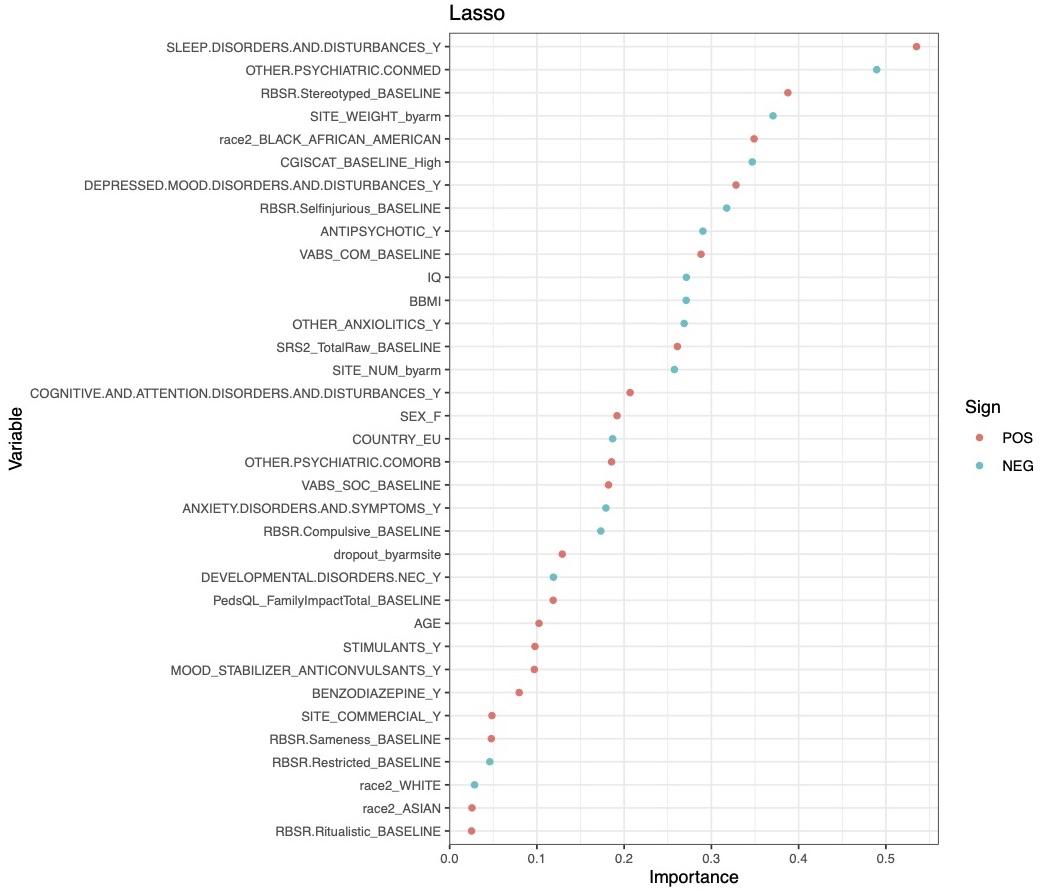


**Figure S4.** Variable selection for change from baseline in CGI-I in the pooled Week 12 cohort. Importance values are based on the estimated standard coefficient with values within +/–1.96 (range set at +/–2) being non-significant, and values outside of –2 and 2 being significant. All the variables were non-significant in this model and hence Step 2 of the analyses were not carried forward for CGI-I. CGI-I Clinical Global Impressions Scale – Improvement, COM communication, EU Europe, IQ intelligence quotient, PedsQL Pediatric Quality of Life Inventory, NEG negative, POS positive, RBS-R Repetitive Behavior Scale – Revised, SOC socialization, VABS Vineland Adaptive Behavior Scales.


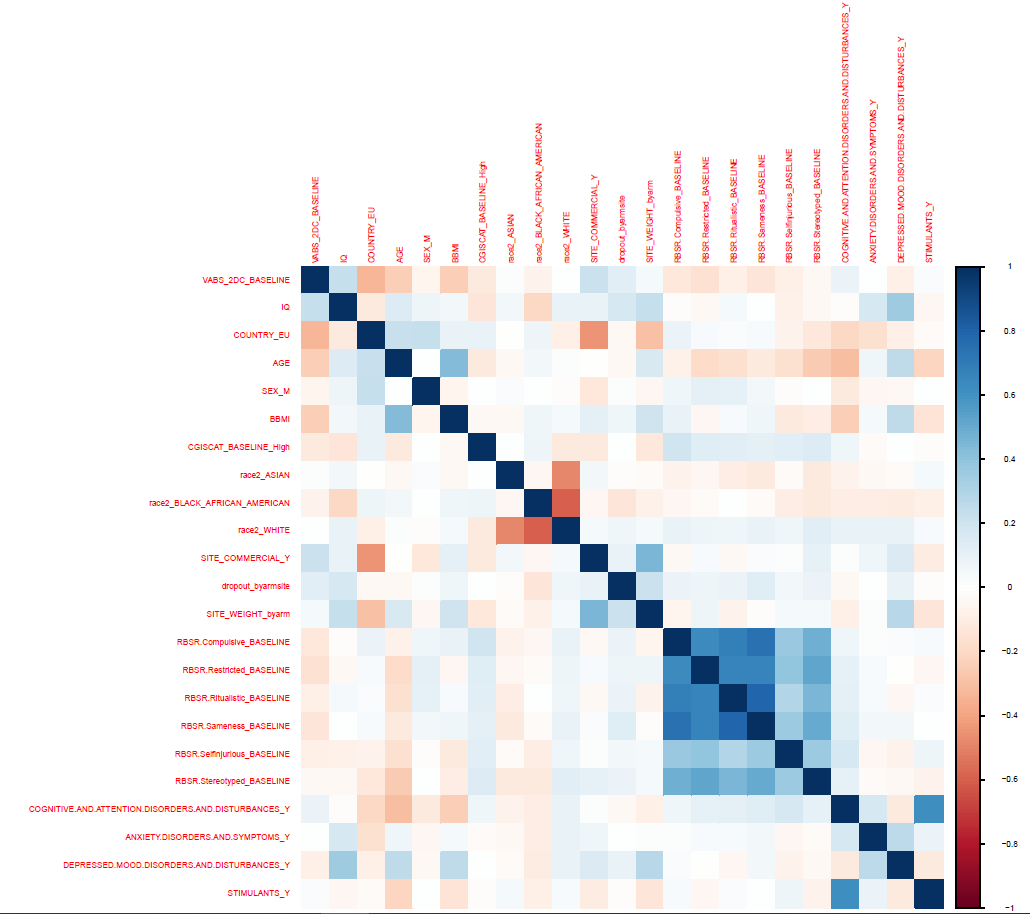


**Figure S5.** Correlations between predictors at baseline. Associations between RBS-R subscales and other predictors were very low, while associations between the subscales themselves were high (the darker the blue box, the higher the correlation). BBMI Baseline BMI, BMI Body mass index, CGISCAT Clinical Global Impressions Scale – Severity, category, EU Europe, IQ Intelligence Quotient, M Male, RBS-R repetitive behavior scale – Revised, VABS 2DC Vineland Adaptive Behavior Scales, Y Yes.

**References**

1. Bolognani F, Del Valle Rubido M, Squassante L, Wandel C, Derks M, Murtagh L, et al. A phase 2 clinical trial of a vasopressin V1a receptor antagonist shows improved adaptive behaviors in men with autism spectrum disorder. Sci Transl Med. 2019;11:eaat7838.

2. Jacob S, Anagnostou E, Hollander E, Jou R, McNamara N, Sikich L, et al. Key insights gained from the balovaptan clinical development program. Mol Autism. 2022;13:25.

3. Jacob S, Veenstra-VanderWeele J, Murphy D, McCracken J, Smith J, Sanders K, et al. Efficacy and safety of balovaptan for socialisation and communication difficulties in autistic adults in North America and Europe: a phase 3, randomised, placebo-controlled trial. Lancet Psychiatry. 2022;9:199–210.

4. Hollander E, Jacob S, Jou R, McNamara N, Sikich L, Tobe R, et al. Balovaptan vs placebo for social communication in childhood autism spectrum disorder: a randomized clinical trial. JAMA Psychiatry. 2022;79:760–9.
